# Computational Tools and Resources for CRISPR/Cas Genome Editing

**DOI:** 10.1016/j.gpb.2022.02.006

**Published:** 2022-03-24

**Authors:** Chao Li, Wen Chu, Rafaqat Ali Gill, Shifei Sang, Yuqin Shi, Xuezhi Hu, Yuting Yang, Qamar U. Zaman, Baohong Zhang

**Affiliations:** 1 Oil Crops Research Institute, Chinese Academy of Agricultural Sciences, Key Laboratory for Biology and Genetic Improvement of Oil Crops, Ministry of Agriculture and Rural Affairs, Wuhan 430062, China; 2 Graduate School of Chinese Academy of Agricultural Sciences, Beijing 100081, China; 3 Department of Biology, East Carolina University, Greenville, NC 27858, USA

**Keywords:** Genome editing, Efficiency and specificity, CRISPR/Cas9, sgRNA, Computational tool, Algorithm

## Abstract

The past decade has witnessed a rapid evolution in identifying more versatile clustered regularly interspaced short palindromic repeats (CRISPR)/CRISPR-associated protein (Cas) nucleases and their functional variants, as well as in developing precise CRISPR/Cas-derived genome editors. The programmable and robust features of the genome editors provide an effective RNA-guided platform for fundamental life science research and subsequent applications in diverse scenarios, including biomedical innovation and targeted crop improvement. One of the most essential principles is to guide alterations in genomic sequences or genes in the intended manner without undesired off-target impacts, which strongly depends on the **efficiency and specificity** of single guide RNA (**sgRNA**)-directed recognition of targeted DNA sequences. Recent advances in empirical scoring **algorithms** and machine learning models have facilitated sgRNA design and off-target prediction. In this review, we first briefly introduce the different features of CRISPR/Cas tools that should be taken into consideration to achieve specific purposes. Secondly, we focus on the computer-assisted tools and resources that are widely used in designing sgRNAs and analyzing CRISPR/Cas-induced on- and off-target mutations. Thirdly, we provide insights into the limitations of available **computational tools** that would help researchers of this field for further optimization. Lastly, we suggest a simple but effective workflow for choosing and applying web-based resources and tools for CRISPR/Cas **genome editing**.

## Introduction

**Figure 1:**
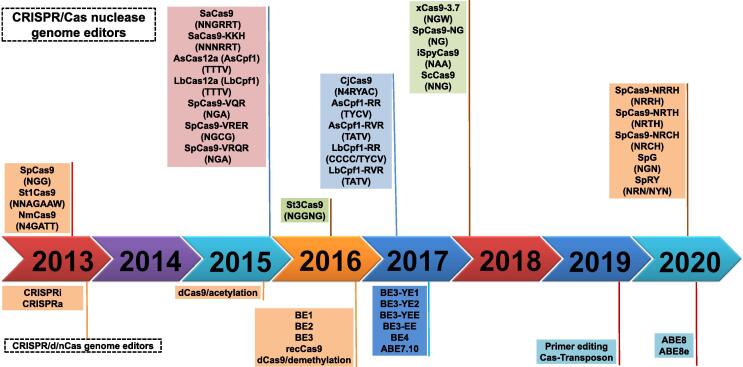
**Overview the brief history for developing the main CRISPR/Cas editors** CRISPR/Cas nuclease genome editors generate site-specific mutations by introducing DSBs on the desired DNA sequences, whereas CRISPR/d/nCas genome editors exert precise modification by fusing with different functional enzymes without inducing a DSB. At present, many Cas nuclease orthologues and variants have been developed with different PAM preferences. To avoid repetition, Cas nucleases with distinct PAM features are organized by timeline. CRISPR/d/nCas editors are generated by combining the targeted binding characteristic of Cas nucleases to the function of various enzyme effectors. For instance, dCas is fused to transcription repressor domains, such as KRAB, for CRISPRi [22]; dCas9 is tethered to transcription activation domains, such as VP64, for CRISPRa; dCas9 is fused to histone acetyltransferase or methylcytosine dioxygenase for epigenetic regulations [23], [24]; dCas9 is fused to transposases and recombinases for targeted integration [20], [21]. The PAM sequences are indicated in brackets, where N represents any base, W represents A/T, R represents A/G, Y represents C/T, H represents A/C/T, and V represents A/C/G. SpCas9-VQR, SpCas9-VRER, SpCas9-VRQR, xCas9-3.7, SpCas9-NG, SpCas9-NRRH, SpCas9-NRTH, and SpCas9-NRCH are different variants of SpCas9; SaCas9-KKH is a variant of SaCas9; AsCpf1-RR and AsCpf1-RVR are variants of AsCpf1; LbCpf1-RR and LbCpf1-RVR are variants of LbCpf1; YE1, YE2, YEE, and EE are narrowed-window CBE variants; ABE7.10, ABE8, and ABE8e are ABE variants. CRISPR/Cas, clustered regularly interspaced short palindromic repeats/CRISPR-associated protein; SpCas9, *Streptococcus pyogenes* Cas9; St1Cas9, *Streptococcus thermophilus* CRISPR1-Cas9; St3Cas9, *Streptococcus thermophilus* CRISPR3-Cas9; NmCas9, *Neisseria meningitides* Cas9; SaCas9, *Staphylococcus aureus* Cas9; AsCas12a, *Acidaminococcus* sp. Cas12a; LbCas12a, *Lachnospiraceae bacterium* Cas12a; CjCas9, *Campylobacter jejuni* Cas9; iSpyCas9, “increased” *Streptococcus pyogenes* Cas9; ScCas9, *Streptococcus canis* Cas9; BE1, first-generation base editor; BE2, second-generation base editor; BE3, third-generation base editor; BE4, fourth-generation base editor; recCas9, Cas9–serine recombinase fusion protein; CBE, cytosine base editor; ABE, adenine base editor; DSB, double-strand break; dCas, dead Cas; nCas, Cas nickase; KRAB, Krüppel-associated box; CRISPRi, CRISPR interference; CRISPRa, CRISPR activation; PAM, protospacer adjacent motif.

The clustered regularly interspaced short palindromic repeats (CRISPR)/CRISPR-associated protein (Cas) system was discovered from the adaptive immune system of bacteria and archaea, which employs ∼ 20-bp RNA CRISPR arrays for guiding Cas nucleases to specifically recognize and cleave the invader’s nucleic acid sequences [1], [2]. In the last decade, this system was developed as a robust genome editing tool to generate sequence-specific mutagenesis at desired genomic sites in a wide range of organisms including both plants and animals [3], [4], [5], [6], [7], [8], [9]. Currently, the CRISPR/Cas genome editing tools have been rapidly modified for further broadening their application potentials [10] (Figure 1). After the Cas9 nuclease, the first discovered Cas nuclease, was used for CRISPR genome editing, other types of Cas nucleases and their orthologues were also proved to have potentials for genome editing. Meanwhile, scientists are also engineering and modifying the existing Cas nucleases to enhance CRISPR/Cas applications. Currently, a variety of CRISPR/Cas-derived genome editors, including base editors and prime editors, provide more options for selecting genome editing tools [11], [12] (Figure 1). Because CRISPR/Cas-based genome editing is precise, robust, and powerful, it has become a revolutionary approach for both foundational and applied research, including clinical CRISPR gene therapy and crop improvement [10], [13], [14].

Although different types of CRISPR/Cas systems exhibit similarities in their genome editing patterns, the recognition and cleavage methods and their underlying machineries are different, which directly determine how to choose the optimal CRISPR/Cas tools for individual experimental purposes. To simplify and accelerate CRISPR/Cas-related research, many laboratories have developed different computational tools and resources for designing single guide RNAs (sgRNAs) and analyzing the genome editing results, including both on- and off-target effects. Currently, CRISPR/Cas tools are not only restricted in genome modification, the high-efficiency binding features of dead Cas9 (dCas9)/Cas9 nickase (nCas9) and their variants allow them to be adapted rapidly for fusing with other functional enzymes to achieve gene regulation, including CRISPR/dCas-mediated gene transcriptional modulation and epigenetic modifications [12], [15], [16], [17], [18], [19], [20], [21], [22], [23], [24]. In this review, we comprehensively summarize our current knowledge of CRISPR/Cas genome editing and the key parameters involved in choosing suitable computational tools. In addition, we systematically summarize the features of several major web-accessible tools for designing sgRNAs and analyzing post-genome editing data; these tools are widely used in both animal and plant genome editing.

## Workflow for performing genome editing experiments

Rapid evolution of CRISPR/Cas genome editing techniques offers more diverse applications that are not just limited to the targeted mutations in desired genomic DNA sequences by inducing double strand breaks (DSBs). Basically, the purpose of applying CRISPR/Cas genome tools is to target and modify a genome sequence, which is subsequently used for identifying gene functions and potential applications, such as human therapeutic purposes and crop genetic improvement [10], [14], [25]. To precisely edit a specific genome sequence by CRISPR/Cas, several key procedures need to be taken into consideration.

Different CRISPR/Cas genome editing techniques have distinct features for achieving certain types of experimental purposes. The common purposes for using CRISPR/Cas tools include: (1) impairing gene functions by creating targeted mutagenesis in their functional domains, which can be achieved by inducing high-frequency DSBs by using the traditional CRISPR/Cas genome editors; (2) remodeling gene roles by precisely modifying specific nucleotide base sequences, which preferably uses base editor and prime editor; and (3) modulating gene expression, in which CRISPR/Cas-based gene activation and repression approaches are usually employed.

**Figure 2:**
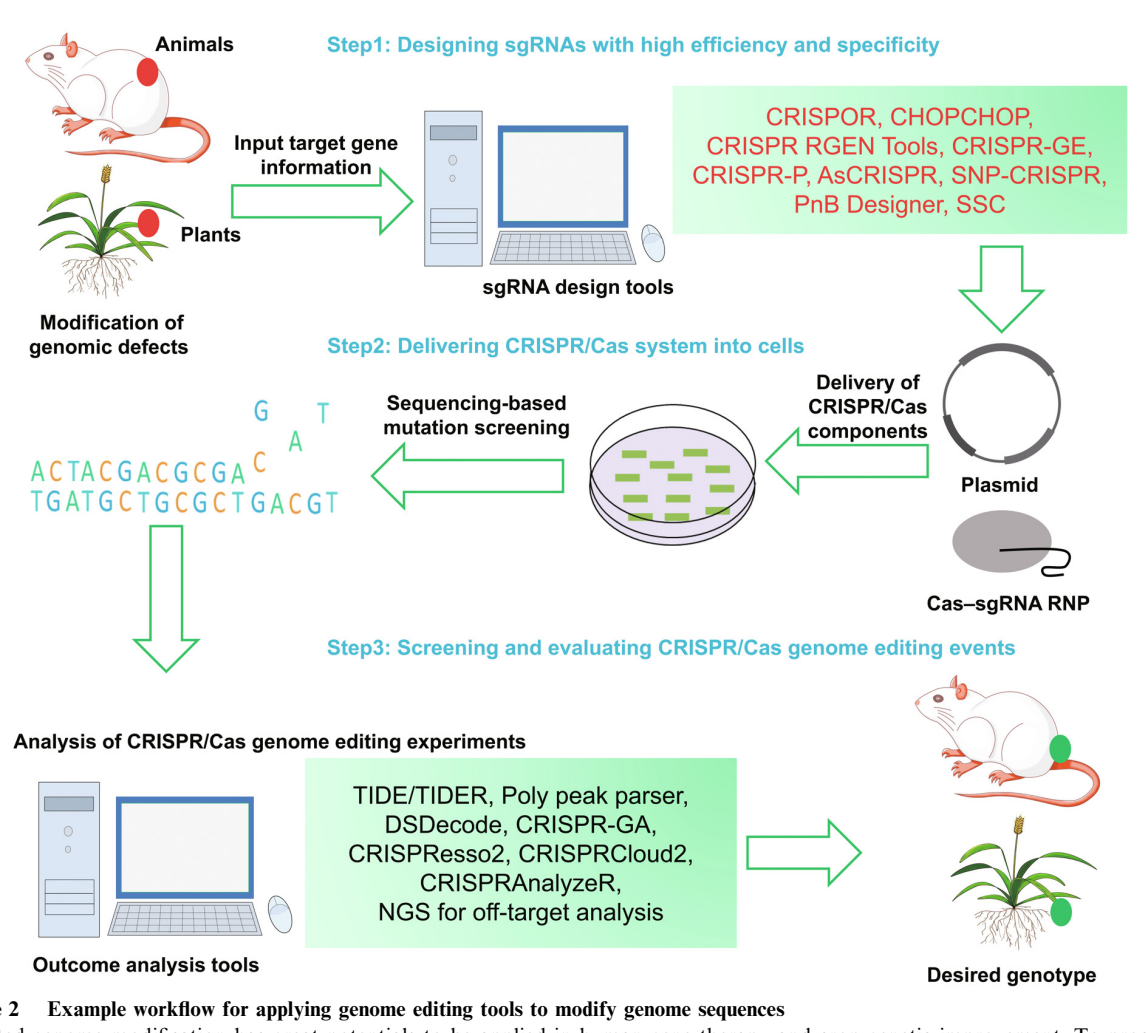
**Example workflow for applying genome editing tools to modify genome sequences** Targeted genome modification has great potentials to be applied in human gene therapy and crop genetic improvement. To proceed a CRISPR/Cas genome editing experiment, the initial step is to design an optimal sgRNA with high efficiency and specificity. On the basis of large-scale empirical data, many algorithm/predictive models have been established and eventually integrated in several web-based applications, such as those shown in upper panel with red color words. Those web-accessible computational tools are designed mainly based on three sets of scoring system, sgRNA efficiency scores, sgRNA specificity scores, and output prediction scores. After performing genome editing experiments, sequencing-based screening will be implemented to evaluate on-target outcomes and off-target effects. To facilitate the efficiency of identifying desired CRISPR/Cas editing events, several web-based resources provide comprehensive computational analysis strategies that meet the needs not only for small-scale genome editing experiments but also for large-scale pooled CRISPR/Cas9 library screening, like those shown in the lower panel with black color words. In addition, many methods and tools have been developed for analyzing outcome off-target effects as listed in Table 2. sgRNA, single guide RNA; NGS, next-generation sequencing; RNP, ribonucleoprotein.

CRISPR/Cas genome editing experiments mainly consist of three major steps (Figure 2): (1) designing sgRNAs to target a gene of interest; (2) choosing an efficient transformation method to deliver the CRISPR/Cas reagents into targeted cells; and (3) screening mutations and analyzing genome editing events. These three steps are extremely important for CRISPR/Cas genome editing. Designing sgRNAs provides a complementary genome site for targeting a specific gene. An ideal sgRNA not only binds to the target sequence with high efficiency but also minimizes the possibility of recognizing other sequence sites that causes off-target effects. Many computational tools have now been developed to design sgRNAs. These web-based computational tools and databases provide a public platform for researchers to identify perfect sgRNAs, and also to predict possible off-target effects.

Delivery of CRISPR/Cas reagents into targeted cells is always required. Without delivering CRISPR/Cas reagents, it is impossible for a sgRNA to bind to the target site and allow Cas enzyme to recognize and edit the specific sequences. There are many transgenic approaches developed for delivering CRISPR/Cas reagents into targeted cells with different purposes. For plants, CRISPR/Cas constructs can be transferred into plant cells by *Agrobacterium*-mediated T-DNA transgene methods, but exogenous fragments can be integrated into plant genomes. CRISPR/Cas ribonucleoprotein (RNP) complex can be used for delivery as well and have been demonstrated in mammalian and plant cells. The sgRNAs and Cas proteins would be degraded after generating mutagenesis, which is beneficial for reducing off-target effects. Many excellent reviews have summarized transgene techniques in detail [26], [27].

After CRISPR/Cas targets the specific sequences, it is necessary to screen the editing events and estimate the potential off-target impacts. Thus, evaluation of the genome editing efficacy is a crucial part of applying CRISPR/Cas genome editing techniques. Successful genome editing should specifically modify the targeted genome sequences without off-target effects on other genome locations. To identify mutation types, many experiment-based methods and high-throughput screening strategies have been developed.

## Best practices for sgRNA design

Efficiency and specificity are two main criteria for CRISPR/Cas genome editing. Efficiency demonstrates how well a sgRNA targets the specific sequence and guides a Cas enzyme to edit the targeted sequences; it is usually presented by the percentage of cells that are edited. Specificity means the CRISPR/Cas editing events are unique or not and whether they cause off-target effects. There are many factors affecting CRISPR/Cas genome editing efficiency and specificity that have been integrated into sgRNA design [28]. The affinity between the RNP complex and the targeted DNA sequences depends on the hybridization of sgRNAs and DNA sequences through sequence complementarity. Previous studies suggest that different binding sites result in huge differences in cleavage efficiency and specificity among different organisms [29], [30], [31], [32]. Several web-accessible databases have been established by collecting sgRNA data from large-scale CRISPR/Cas experiments [33], [34], [35], [36], [37] (Table 1). Based on the analysis, these databases not only provide practical resources for sgRNA selection but also reveal the key factors that affect sgRNA efficacy and specificity, which would facilitate the further optimization of sgRNA design.

**Table 1:** **Commonly used sgRNA design tools and databases**

**Name**	**Organism**	**Cas nuclease**	**Major feature**	**Database or web server**	**Website**	**Refs.**
CRISPOR	> 100 species	> 30 Cas9 orthologues and Cas variants	Designing, evaluating, and cloning guide sequences for the CRISPR/Cas9 system; providing primers for vector construction; indicating mismatch number; and linking off-target to genome browser	Web server	https://crispor.tefor.net/	[38]
CHOPCHOP	> 100 species	Cas9, Cas12, Cas13, and TALEN	Providing multiple predictive models; visualizing genomic location of targets and genes; and providing primers	Web server	https://chopchop.cbu.uib.no/	[46], [47]
CRISPR RGEN Tools	> 100 species	> 20 Cas9 orthologues and Cas variants	Providing multiple predictive models; downloadable and standalone; and predicting potential off-target number via Cas-OFFinder, and out-of-frame scores via Microhomology-Predictor	Web server	https://www.rgenome.net/cas-designer/	[84], [93]
E-CRISP	> 50 species	SpCas9	Feasibly creating genome-scale libraries; downloadable; and frequently updated	Web server	https://www.e-crisp.org/E-CRISP/index.html	[49]
GUIDES	Human and mouse	SpCas9	Feasibly designing CRISPR knockout libraries; downloadable; and step-by-step	Web server	https://guides.sanjanalab.org/ and https://github.com/sanjanalab/GUIDES	[11]
CRISPRscan	> 10 species	Cas9 and Cas12	Designing sgRNAs for protein-coding genes; ready-to-inject sgRNA sequence; tracks for genome browser; and searching whole-genome off-target impacts	Web server	https://www.crisprscan.org/	[45]
CCTop	> 100 species	> 10 Cas9 orthologues and Cas variants	Searching for single and multiple queries; indicating mismatch number; predicting off-target impacts; and predicting sgRNA efficiency using CRISPRater with custom *in vitro* transcription selection	Web server	https://cctop.cos.uni-heidelberg.de/	[39]
CRISTA	> 100 species	SpCas9	Providing machine learning framework, including DNA/RNA bulge genomic context and RNA thermodynamics; detecting off-targets; and ranking targets	Web server	https://crista.tau.ac.il/	[56]
DeepCRISPR	Human	SpCas9	Incorporating epigenetic information; and predicting off-target impacts	Web server	https://www.deepcrispr.net/	[57]
DRSC Find CRISPRs	*Drosophila*	SpCas9	Providing off-target stringency from 3 to 5 mismatches; and separating target region and potential off-targets by different tracts	Web server	https://www.flyrnai.org/crispr/ https://www.flyrnai.org/crispr3/web/	[72]
EuPaGDT	Eukaryotic pathogens	> 10 Cas9 orthologues and Cas variants	Providing wide compatibility for eukaryotic pathogen genomes	Web server	https://grna.ctegd.uga.edu/	[73]
WU-CRISPR	Human and mouse	SpCas9	Providing machine learning algorithm trained by experimental data; providing custom sequence between 26 bp and 30,000 bp with one sequence per time; and downloadable results	Web server	https://crispr.wustl.edu/	[155], [156]
GPP sgRNA Designer	Human, mouse, and rat	SpCas9, SaCas9, and AsCpf1	Inputting up to 200 transcript IDs or gene IDs; maximizing on-target activity and minimizing off-target activity; and scoring on-targeting efforts	Web server	https://portals.broadinstitute.org/gpp/public/analysis-tools/sgrna-design	[48]
CRISPR-GE	> 40 plant species	SpCas9, FnCpf1, and AsCpf1	Providing software toolkits, primer design for vector construction, on-target amplification, and PCR sequencing result analysis	Web server	https://skl.scau.edu.cn/	[94]
CRISPR-P	49 plant species	> 14 Cas9 and variants	Supporting wide range of plant species; providing on-target and off-target scoring; and providing gRNA sequence analysis	Web server	https://crispr.hzau.edu.cn/CRISPR2/	[95], [96]
CRISPR-PLANT V2	7 plant species	SpCas9	Supporting main model and crop plant species; providing selection of chromosome and locations with clear instruction	Web server	https://www.genome.arizona.edu/crispr2/	[157]
CRISPRz	Zebrafish, human, and mouse	SpCas9	Providing specific for a wide variety of cell lines and organisms including zebrafish; and providing validated sgRNA database	Web server	https://research.nhgri.nih.gov/CRISPRz/	[82]
CRISPRlnc	10 species	SpCas9	Providing downloadable validated sgRNA database for lncRNAs	Database	https://www.crisprlnc.org/	[81]
FORECasT	Human	SpCas9	Predicting the mutational outcomes	Web server	https://partslab.sanger.ac.uk/FORECasT	[87]
AsCRISPR	Human and mouse	SpCas9, AsCpf1, AaCas12b, CasX, and variants	Designing sgRNAs for allele-specific genetic elements	Web server	https://www.genemed.tech/ascrispr/ascrispr	[98]
SNP-CRISPR	9 plant and animal species	NGG and NAG PAM	Designing sgRNAs for targeting SNPs or Indel variants	Web server	https://www.flyrnai.org/tools/snp_crispr/web/	[99]
SSC	N/A	Cas9	For both CRISPR knockout and CRISPRa/CRISPRi	Web server	https://cistrome.org/SSC/	[35]
DeepHF	N/A	SPCas9 and Cas9HF	gRNA designer and efficiency prediction	Web server and database	https://www.DeepHF.com/	[158]
PnB Designer	6 species	Cas9	Designing pegRNAs for prime editors and sgRNAs for base editors	Web server	https://fgcz-shiny.uzh.ch/PnBDesigner/	[99], [100]
inDelphi	Human	SpCas9	Predicting the mutational outcomes	Web server	https://www.crisprindelphi.design/	[86]

*Note*: Cas, CRISPR-associated protein; CRISPR, clustered regularly interspaced short palindromic repeats; CRISPRa, CRISPR activation; CRISPRi, CRISPR interference; gRNA, guide RNA; lncRNA, long non-coding RNA; pegRNA; prime editing guide RNA; sgRNA, single guide RNA; TALEN, transcription activator-like effector nuclease; N/A, not available.

To systemically characterize the relationship between sgRNA features and cleavage efficiency, Zhang and coworkers assessed more than 700 sgRNA variants and over 100 potential target sites in human cells [33]. Their results suggested that the total number, position, and distribution of mismatched bases were crucial to determine the cleavage activity of CRISPR/Cas9 targets [33]. In addition, a mismatched single-base located in the protospacer adjacent motif (PAM)-proximal region is more sensitive than the PAM-distal counterparts [33]. To refine sgRNA efficacy and its prediction, Labuhn and colleagues employed fluorescent reporter knockout assays to test the target efficacies of 430 sgRNAs; based on their experimental results, they developed a linear model-based discrete system, called CRISPRater, for predicting sgRNA efficiency [36]. Currently, this algorithm has been integrated with other sgRNA designing programs, such as CRISPOR [38] and CCTop [39].

### Effect of nucleotide composition and location on sgRNA design

The nucleotide composition of a sgRNA, particularly GC content, is essential to determine its efficiency and specificity. One of the most important applications of CRISPR/Cas tools is to perform whole-genome screening for gene functional analysis [31], which also provides important information for uncovering nucleotide preference of sgRNAs. Based on analyzing the data of 1841 sgRNAs designed for targeting endogenous mouse and human genes, Doench and colleagues developed a predictive model (named Rule Set 1, which is based on sgRNA sequence features) to clarify general rules for designing highly active sgRNAs [40]. After quantification of the sequence features correlated with the activities of sgRNAs, they found that the GC content of a sgRNA did not display a positive correlation with the sgRNA activity in genome editing; both high and low GC contents of sgRNAs led to less efficient genome editing [40]. A similar rule was also identified in performing genome-scale functional screens using human cells and zebrafish [31], [41]. Additionally, several large-scale datasets suggest that the type of nucleobase is important for sgRNA activity [40], [42]. The nucleotide at the position 20, located immediately upstream of PAM, is a key determinant. Guanine was highly favorable whereas cytosine was strongly unfavorable [31], [40], [41]. In contrast, the position 16, the last nucleotide of the seed region, preferred cytosine over guanine [40], [42]. Theoretically, the transcription of sgRNAs relies on RNA polymerase III that recognizes uracil-rich sequences for termination [43], [44]. The uracil-rich sequence structure might lead to early termination of sgRNAs and then impair expression [42]. Thus, sgRNA sequences with thymine-rich nucleobase are not favorable at their 3′ end region. Additionally, adenine is preferable in the middle of a sgRNA, whereas cytosine has negative effects at the position 3 [31], [40].

Zebrafish is an ideal model organism for performing large-scale analysis of sgRNA activity. To dissect the sgRNA molecular features affecting the efficacy of CRISPR/Cas9 *in vivo*, a sgRNA pool was constructed by introducing 1280 sgRNAs to target 128 genes in the zebrafish genome [45]. The researchers found that sgRNA stability *in vivo* plays a critical role in determining sgRNA activity. The formation of a guanine-quadruplex structure, which contains at least eight guanines, can significantly increase sgRNA stability. Additionally, several sequence features were identified by statistical analysis of the most efficient sgRNAs, such as guanine enrichment in the region of positions 1–14, cytosine enrichment between the position 15 and the position 18, and overall depletion of thymidine and adenine except the positions 9 and 10 [45]. Taken together, a linear regression-based predictive sgRNA-scoring algorithm, named CRISPRscan (http://CRISPRscan.org), was proposed for detecting the most active sgRNAs *in vivo* [45]. The CRISPRscan model is also implemented in other web-based sgRNA design tools, such as CHOPCHOP [46], [47] and CRISPOR [38].

Given the hypothesis that sgRNA activity could be influenced by several other features, such as the position-independent nucleotides, the location of the target sites in the gene, and the thermodynamic property of a sgRNA, the Rule Set 1 predictive model was further improved by integrating new prediction algorithms and generated “Rule Set 2”. It employs the improved algorithms for on- and off-target activity prediction, and the gradient-boosted regression tree model with the augmented feature set trained on the combined dataset, which is used not only for sgRNA libraries for general genome editing purposes (gene knockout and knockin) but also for CRISPR activation (CRISPRa) and CRISPR interference (CRISPRi) [37]. The Rule Sets 1 and 2 were widely implemented in many websites and computational tools for designing sgRNAs, including CHOPCHOP [46], [47], CRISPOR [38], GPP sgRNA Designer [48], and E-CRISP [49].

Some other factors also affect Cas nuclease binding and cleavage. It has been suggested that both sequence composition and locus accessibility are important to determine sgRNA activity, which subsequently influence the sgRNA design tools, such as sgRNAScorer [50], [51]. Additionally, chromatin accessibility [52], [53], [54], [55] and asymmetric sgRNA–DNA interactions also affect CRISPR/Cas cutting specificity [37], [56]. Currently, many groups have integrated these algorithms into their web-based applications, such as DeepCRISPR, CRISTA [56], [57], predictSGRNA [58], and uCRISPR [59]. GuidePro is a two-layer ensemble predictor for sgRNA efficiency prediction that enables the integration of multiple factors for the prioritization of sgRNAs for gene knockout [60].

### Designing prime editing guide RNAs for prime editing

Prime editing is a new application of CRISPR/Cas technology in which a small-sized genetic sequence is altered without requiring a donor DNA template. In prime editing system, a prime editing guide RNA (pegRNA) is used to replace the traditional sgRNA, which contains a primer binding site (PBS) and a reverse transcriptase (RT) template sequence. After nCas9 cuts a target DNA sequence, the PBS sequence will be elongated and inserted into the original DNA sequence for DNA replacement [61]. Thus, prime editing can be used to repair any nucleotide error without a DNA template. Due to these advantages, prime editor has huge potentials for genome editing. However, evaluation of prime editing efficiency is time- and lab-intensive. To solve this problem, Kim and colleagues used deep learning to create a precise computational model for measuring the efficiency of pegRNAs based on high-throughput evaluation of 54,836 pegRNA–target pairs in human cells [62]. More importantly, this computational tool and resources can be found in their publicly available website http://deepcrispr.info/DeepPE/.

### Off-target consideration

One of the main concerns about sgRNA design is off-target effects that are normally generated by unexpected cleavage at genomic sites similar to the target sequences [33], [63]. Thus, traditional short sequence alignment tools, such as Burrows-Wheeler Alignment Tool (BWA) and Bowtie [64], [65], [66], have been used to predict potential off-target sites [38], [49]. Given that BWA and Bowtie are originally designed for aligning short DNA reads to large reference genomes [64], [65], there are several innate defects for predicting off-target effects. For instance, CRISPR/Cas has been suggested to tolerate more mismatches than traditional BWA or Bowtie alignment allows [33], [67], [68]. Additionally, nucleotide positions are important for target specificity, and atypical PAM could be recognized by CRISPR/Cas9 as well [33], [37]. To overcome these problems, many improved off-target prediction tools have been reported. For example, CCTop can predict potential off-target sites with four mismatches differently distributed in the targeted genomic sites [39], and Cas-OFFinder is not limited by the number of mismatches and allows variations in PAM sequences [67].

To predict off-target sites more accurately, several computational models were built based on large amounts of experimental data. After evaluating more than 100 predicted genomic off-target loci in two human embryonic kidney cell lines [33], several rules were proposed to minimize off-target effects, including that (1) the potential off-target sequences should not be followed by a PAM with either a 5′-NGG or 5′-NAG sequence, and (2) the minimum mismatches between sgRNA and potential off-target sites should be limited to 3 nt and at least two mismatches are better in the proximal PAM region. These rules have been implemented in their specificity score tool, termed MIT, which has subsequently been implemented in web-accessible applications, such as CHOPCHOP [46], [47] and CRISPOR [38]. Another commonly used specificity score tool is Cutting Frequency Determination (CFD), proposed by Doench and colleagues [37]. In addition to mismatch position of sgRNA and atypical PAM effect, the identities of mismatched nucleotides and insertion and deletion (indel) variants can significantly affect sgRNA activity. CFD has been shown to predict most off-target sites and exhibit better performance than MIT and CCtop by using GUIDE-seq, an unbiased experimental method for detection of sgRNA off-target effects [69]. CFD has been implemented in CRISPOR, GPP sgRNA Designer, GUIDES, and other web-related tools.

Currently, there are many computational programs for designing sgRNAs and predicting their genome editing efficiency and specificity. To comprehensively benchmark these techniques and tools, several available on-target design tools, genome-wide off-target cleavage site (OTS) detection techniques, and *in silico* genome-wide OTS prediction tools have been systematically evaluated [70], [71]. A one-stop platform, named integrated Genome-Wide Off-target cleavage Search platform (iGWOS), was constructed by integrating these available OTS prediction algorithms and datasets [70], [71].

## Web-based tools and resources available for designing sgRNAs

The growing application of CRISPR/Cas techniques provides more data to optimize computational analysis models. As shown in Table 1, a large number of available sgRNA design tools have been compared and the majority of them displayed different features.

Because genetic and epigenetic features of the genome are essential to sgRNA efficacy, many comprehensive sgRNA design websites are constructed for diverse genomes, such as CHOPCHOP, CRISPOR, CRISPR RGEN Tools, and E-CRISP. Some are compatible with dozens or even hundreds of organisms (Table 1). However, other tools are restricted to a certain type of genome background. For instance, CRISPR-PLANT, CRISPR-P, and CRISPR-GE are online sgRNA design resources that mainly serve plant species. DRSC Find CRISPRs was designed for genome editing of *Drosophila* [72]. EuPaGDT is a tailored website tool for eukaryotic pathogens [73]. In contrast to the comprehensive websites that only offer sgRNA design services, these organism-specialized tools usually provide empirical CRISPR/Cas vectors and protocols that are very useful for wet lab experiments. Moreover, CRISPy-web implements sgRNA design with a user-provided microbial genome [74]. Thus, based on individual research objectives, the first step is always to design an appropriate sgRNA by selecting a suitable sgRNA design tool.

Selecting a genome editing system also depends on the experimental purpose. Constructing genome-scale CRISPR/Cas9 knockout libraries has been achieved in certain organisms, such as human cells [31], [34], [75], mouse [76], [77], zebrafish [78], and rice [79], [80]. To this end, Graphical User Interface for DNA Editing Screens (GUIDES) provides a website application for constructing genome-wide CRISPR/Cas-mediated mutation libraries in human and mouse genomes [11]. Additionally, CRISPRlnc and CRISPRz web tools are established by collecting experimentally validated sgRNAs generated from large-scale mutagenesis data and published sources [81], [82], which can be directly chosen for subsequent experiments. However, for small-scale genome editing experiments, PAM requirements should be one of the most important limitations for designing sgRNAs. Some websites only support SpCas9, whereas others have many Cas nuclease options and relatively broad ranges of PAM variants available for diverse experimental purposes. Additionally, certain tools, such as CHOPCHOP, provide an “Option” menu that can customize PAM types.

As summarized in the aforementioned discussion, many predictive models and scoring algorithms have been developed for predicting sgRNA specificity and efficiency, which may have distinct predictive scoring system. CRISPOR and CHOPCHOP integrate multiple scoring models into their web tools. For example, ten efficiency scores and two specificity scores have been combined in CRISPOR tool; CHOPCHOP employs six efficiency scores and two specificity scores.

Predicting CRISPR/Cas outcomes is a relatively new development for increasing the accuracy of sgRNA design. Non-homologous end-joining (NHEJ) is a central mechanism for repairing CRISPR/Cas-generated DSBs. Since NHEJ simply rejoins break ends together without using a homologous sequence for guidance template, this error-prone repair approach has been considered as the major method for inducing indel mutations at the DSB sites. Previous studies have demonstrated that NHEJ-mediated error-prone repair is nonrandom and strongly biased by short and homologous sequences around the DSBs, termed microhomology mediated end joining (MMEJ) [83], [84], [85]. FORECasT and inDelphi are two recommended CRISPR/Cas predictive tools that were developed by training with large-scale experimental data [86], [87].

Because human therapeutic treatments and crop genetic improvement are two main application areas of CRISPR/Cas technology, several web-based tools, which are commonly used in animal and plant genome editing, are recommend below.

### CRISPOR

CRISPOR provides multiple tools that include efficiency prediction, specificity prediction, and a primer design tool for vector construction as well as on-target and off-target detection. CRISPOR incorporates almost all empirical algorithms for predicting efficiency, such as Rule Set 2 [37], [40], CRISPRscan [45], Wang et al. [31], Chari et al. [51], and Xu and coworkers [35]. They also apply “deepCpf1” and “Najm et al.” to predict Cas12a and SaCas9 efficiencies [88], [89], [90], respectively. The predicted results are well visualized by these models. For specificity prediction, CRISPOR includes MIT and CFD that are two mainstream specificity prediction tools. CRISPOR also integrates two CRISPR/Cas outcome predictive models, out-of-frame score and frameshift ratio [84], [85], to further reduce cutting efficiency. In addition, several critical factors such as the GC content and the type and number of mismatches (0–4 nt) are labeled in the results. CRISPOR covers hundreds of organisms. Different nucleases and PAM types are also available for selection. These features allow the majority of researchers to use CRISPOR for designing different CRISPR/Cas genome editing experiments.

### CHOPCHOP

CHOPCHOP is also a comprehensive website for sgRNA design. Both CRISPR/Cas and transcription activator-like effector nuclease (TALEN) systems are supported by CHOPCHOP. Additionally, CHOPCHOP provides various targeting systems, such as knockout, knock-in, gene activation, and gene repression. Similar to CRISPOR, CHOPCHOP also provides multiple predictive models, and the user can choose one of them to predict cutting specificity and efficiency. In addition, CHOPCHOP has a “Custom PAM” option that is convenient for choosing different PAM sequences. It has been reported that cell types may affect the DSB repair pathway and then influence CRISPR/Cas genome editing outcomes [91], [92]. Several cell types, including mESC, U2OS, HEK293, HCT116, and K562, are optional in the CHOPCHOP website for accurate outcome prediction. It is also important that CHOPCHOP is compatible with more than 200 genomes. It allows researchers to design sgRNAs in a specific region of a gene, such as 5′ UTR, 3′ UTR, promoter, or the coding region.

### CRISPR RGEN Tools

CRISPR RGEN Tools is a CRISIPR/Cas library platform that contains multiple sgRNA design tools. For example, CRISPR RGEN Tools employs Cas-designer for conventional CRISPR/Cas nucleases, BE-Designer for CRISPR base editing, and PE-Designer for CRISPR prime editing [93]. In addition, PE-Designer only allows for SpCas9; both Cas-designer and BE-Designer have wide PAM compatibility. More than 100 organisms are well organized in those three tools. Microhomology-Predictor is an outcome-predictive tool that introduces out-of-frame score algorithm to evaluate potential in-frame deletions caused by the MMEJ repair approach [84]. In addition to CRISPR/Cas, this tool also supports other programmable nucleases, such as zinc finger nucleases (ZFNs) and TALENs, and an out-of-frame score over 66 is recommended. Thus, a user can utilize those tools to implement different experimental purposes; it is also helpful for designing sgRNAs with high accuracy.

### CRISPR-GE

CRISPR-GE is a web-based tool for designing sgRNAs in plants [94]. CRISPR-GE covers 41 plant genomes, including several agriculturally important crops, such as rice (*Oryza sativa japonica*), corn (*Zea mays*), and grape (*Vitis vinifera*). This tool also includes multiple Cas nucleases, such as SpCas9, FnCas12a, and AsCas12a, for helping the users to design sgRNAs for different CRISPR/Cas systems. Additionally, CRISPR-GE provides a “User defined” option that allows the users to customize PAM sequences (including 5′ and 3′ PAMs) and the length of target sites. CRISPR-GE provides warning notes for indicating “bad site”, such as very low or very high GC contents, poly-T site(s), and contiguous base-pairing with a sgRNA. CRISPR-GE implements CFD model to predict the specificity of a target site. CRISPR-GE also provides a primer design tool to assist vector construction and mutant detection.

### CRISPR-P

CRISPR-P is another web-based tool for designing sgRNAs for plants [95], [96], which covers 75 plant genomes and the majority of them are important grain crops. Compared with CRISPR-GE, there are more CRISPR/Cas PAM types available in CRISPR-P, which include NGG (SpCas9), NNAGAAW (St1Cas9), N_4_GMTT (NmCas9), NNGRRT (SaCas9), and NG (xCas9). Additionally, CRISPR-P allows the users to choose *U3* or *U6* sgRNA promoter-driven expression cassettes for designing sgRNAs. The users can input gene ID/name, position on scaffold and chromosome, and fasta format sequences for submitting data. CRISPR-P implements Rule Set 1/2 and CFD to predict on-target and off-target effects. The sgRNA predictive outputs are well visualized, which includes sgRNA GC content, restriction endonuclease site, secondary structure of sgRNA [97], and microhomology score [84].

### AsCRISPR

AsCRISPR is a comprehensive web tool for designing sgRNAs for allele-specific genome elements, which can be used to discriminate between alleles. This tool is specifically designed for targeting dominant single nucleotide variants (SNVs) retrieved from ClinVar and OMIM databases [98]. In this publicly available web tool, several Cas enzymes, such as SpCas9, AsCas12a, and Cas12v, as well as CasX and their variants, can be selected. Currently, this web tool is only for targeting SNVs in the human and mouse genomes.

### SNP-CRISPR

SNP-CRISPR is a web-based computational program for designing sgRNAs based on public variant datasets or user-identified variants [99]. It can be used for both model species and non-reference genomes as well as across varying genetic backgrounds, particularly for SNP-containing alleles. SNP-CRISPR also calculates the efficiency and specificity scores for sgRNA designs targeting both the variants and the reference.

### PnB Designer

PnB Designer is a web-based tool for designing sgRNAs for both prime and base editors, two newly developed CRISPR/Cas genome editors [100]. PnB Designer design sgRNAs for both single and multiple genome targets on several different plant and animal species.

### Sequence scan for CRISPR

Sequence scan for CRISPR (SSC; https://cistrome.org/SSC/) is one online web server for scanning sgRNA spacer [35]. It is not only for designing sgRNAs for CRISPR knockout but also for CRISPR inhibition or activation with sgRNA efficiency prediction.

In addition to academic-developed publicly available computational tools, certain CRISPR companies have also developed several useful computational tools and resources for the public. These design tools include, but are not limited to: IDT (https://www.idtdna.com/site/order/designtool/index/CRISPR_CUSTOM), Horizon (https://horizondiscovery.com/en/ordering-and-calculation-tools/crispr-design-tool), and Synthego (https://www.synthego.com/products/bioinformatics/crispr-design-tool).

## Best practice for downstream analysis and tools/resources available for performing downstream analysis

To identify desired genome editing events after CRISPR/Cas genome editing experiments, many experiment-based methods and computational tools have been developed for detecting the indels induced by genome editing enzymes in the targeted sequences. In 1995, Mashal and colleagues developed a method that frequently determines the level of activity for a sgRNA in hetero-duplexed DNA (hdDNA) [101]. In this assay, reagents are transfected into the cells; genomic DNA surrounding the target locus is amplified by using polymerase chain reaction (PCR). Then, the PCR products are denatured and re-complexed under heating and then subsequent slow cooling. If an aberrant NHEJ event occurred, a heteroduplex forms between amplicons of different length in mutant and wild-type amplicons. These amplicons lead to DNA distortion, which is recognized and cleaved by T7 endonuclease I (T7E1). This method has been widely adopted to test CRISPR/Cas9 genome editing events. However, the accuracy of the T7E1 enzyme is questioned due to the low dynamic range and the requirement of hetero-duplex formation, which lead to incorrect prediction of sgRNA activity [102].

### Decoding Sanger sequencing of on-target sites

To enable easy quantification of CRISPR/Cas9 genome editing products, several new methods have been developed by directly decoding Sanger sequencing data (Table 2). For example, tracking of indels by decomposition assesses (TIDE) is a decomposition algorithm that is able to precisely determine the indel spectrum and frequency of targeted mutations generated by CRISPR/Cas9 genome editing [103]. It is a very simple and effective method to assess the efficiency of well-performing sgRNAs. It only requires standard molecular biology reagents and involves three steps, including a standard PCR reaction, Sanger sequencing, and decoding raw sequencing data by the TIDE web tool. The algorithms accurately reconstruct the spectrum of indels from the sequence traces. The web tool reports the identity of the detected indels and their frequencies [104]. Moreover, it is highly effective to predict indels with all sizes in sample clones as well as tracing indels in heterozygotes [102]. TIDE has been further designed to decompose the sequence data produced by template-directed CRISPR/Cas genome editing experiments [105]. Since the majority of CRISPR/Cas-induced mutations in plants are biallelic (two distinct variations), homozygous (two identical mutations), and heterozygous (wild-type/single mutation) [106], Liu and colleagues established a web-based tool, termed DSDecode, to automatically decoding the superimposed sequencing chromatograms of CRISPR/Cas PCR products [107].

**Table 2:** **List of CRISPR/Cas outcome analysis tools**

**Type**	**Name**	**Description**	**Website**	**Web server or standalone tool**	**Ref.**
Decoding Sanger sequencing of on-target sites	TIDE	Quantifying non-templated CRISPR/Cas9 mutations	https://tide.nki.nl	Web server	[103]
	TIDER	Quantifying the indels of templated CRISPR/Cas9 editing	https://tide.nki.nl	Web server	[105]
	EditR	Quantifying base editing results	https://baseeditr.com/	Standalone tool	[159]
	Poly peak parser	Quantifying heterozygous indels	http://yost.genetics.utah.edu/software.php	Standalone tool	[160]
	DSDecode	Automatically decoding the sequencing chromatograms	http://skl.scau.edu.cn/dsdecode/	Standalone tool	[107]
NGS evaluation of targeted amplicon sequences	BATCH-GE	Detecting the on- and off-target impacts by analyzing deep sequencing data and calculating mutagenesis efficiencies	https://github.com/WouterSteyaert/BATCH-GE	Standalone tool	[115]
	CRISPR-GA	Quantifying and characterizing indels and homologous recombination events	https://crispr-ga.net	Web server	[108]
	CRISPResso2	Enabling the users to analyze, visualize, and compare CRISPR outputs from hundreds of experiments using batch functionality	https://crispresso.pinellolab.partners.org/submission	Web server	[110]
	Cas-Analyzer	Measuring the frequencies of mutations induced by CRISPR/Cas9 and other programmable nucleases for NGS data analysis	http://www.rgenome.net/cas-analyzer/	Web server	[109]
	CRIS.py	Providing a Python-based software to analyze NGS data for both knockout and knock-in (multiple users specified) modifications from one to thousands of samples at once	https://github.com/patrickc01/CRIS.py; https://s.stjude.org/video/player.html?videoId=6000021936001	Standalone tool	[111]
	CRISPRpic	Providing precise mutation calling and ultrafast analysis of the sequencing results	https://github.com/compbio/CRISPRpic	Web server	[161]
	CRISPR-DAV	Providing high-throughput analysis of amplicon-based NGS data	https://github.com/pinetree1/crispr-dav	Standalone tool	[162]
	GNL-Scorer	Combining optimal datasets, models, and features, to address the cross-species problem	https://github.com/TerminatorJ/GNL_Scorer	Standalone tool	[114]
	CrispRVariants	Quantifying Sanger sequencing and high-throughput amplicon sequencing	https://www.bioconductor.org/packages/CrispRVariants	Standalone tool	[112]
NGS evaluation of pooled CRISPR/Cas9 libraries	CRISPRCloud2	Providing accurately mapping short reads to CRISPR library; statistically aggregating the information across multiple sgRNAs targeting the same gene; providing a user-friendly data visualization and query interface; easy linking with other tools and bioinformatic resources for target preference	https://crispr.nrihub.org	Web server	[125]
	CRISPRAnalyzeR	Featuring with eight hit calling strategies including DESeq2, MAGeCK, edgeR, sgRSEA, Z-Ratio, Mann-Whitney test, ScreenBEAM, and BAGEL; exploring the pooled CRISR/Cas9 screens	https://www.crispr-analyzer.org; https://www.github.com/boutroslab/CRISPRAnalyzeR	Standalone tool	[123]
	PinAPL-Py	Providing a comprehensive workflow covering quality control, automated sgRNA sequence extraction and alignment, sgRNA enrichment/depletion analysis, and gene ranking	https://pinapl-py.ucsd.edu	Web server	[124]
	MAGeCK	Providing analysis of large-scale screens	https://bitbucket.org/liulab/mageck/src/master/	Standalone tool	[117]
	MAGeCK-VISPR	Providing analysis of large-scale screens	https://bitbucket.org/liulab/mageck-vispr	Standalone tool	[163]
	BAGEL	Providing analysis of large-scale screens	https://bagel-for-knockout-screens.sourceforge.net/	Standalone tool	[121]
	HiTSelect	Providing analysis of large-scale screens	https://github.com/diazlab/HiTSelect	Standalone tool	[119]
	caRpools	Providing analysis of large-scale screens	https://github.com/boutroslab/caRpools	Standalone tool	[118]
	CHANGE-seq	Measuring the genome-wide activity of Cas9		Standalone tool	[130]
	ScreenBEAM	Providing analysis of large-scale screens	https://github.com/jyyu/ScreenBEAM	Standalone tool	[120]
	CERES	Providing CRISPR screen analysis	https://depmap.org/ceres/	Standalone tool	[164]
	PBNPA	Providing analysis of large-scale screens	https://cran.r-project.org/web/packages/PBNPA/	Standalone tool, database	[122]
NGS evaluation of off-target effects	DISCOVER-Seq	Detecting unbiasedly off-targets by precise tracking of MRE11; exploring molecular nature of Cas activity in cell with single-base resolution	N/A	Standalone tool	[129]
	HTGTS	Providing robust detection of DSBs generated by engineered nucleases based on their translocation to other endogenous or ectopic DSBs	weblogo.berkeley.edu	Standalone tool	[165]
	IDLVs	Detecting off-target cleavages with a frequency as low as 1%; providing frequent off-target sites up to 13 mismatches between the sgRNA and its genomic target	N/A	Standalone tool	[127]
	BLESS	Mapping DNA DSBs at nucleotide resolution by detecting telomere ends, *Sce* endonuclease-induced DSBs, and complex genome-wide DSB landscapes	N/A	Standalone tool	
	BLISS	Measuring the location and frequency of DSBs in genome by direct labeling of DSBs in fixed cells or tissues; quantifying DSBs through unique molecular identifiers; low input requirement	N/A	Standalone tool	[166]
	GUIDE-seq	Providing unbiased and global detection of DSBs induced by CRISPR RNA-guided nucleases	N/A	Standalone tool	[69]
	GOTI	Providing comparison of edited and non-edited cells distinguished by Cre-*loxP* recombination system	https://github.com/sydaileen/GOTI-seq	Standalone tool	[167]
	SITE-Seq	Identifying off-targets *in vitro* by integrating biochemical assay to increase the enrichment of CRISPR/Cas cleavage fragments	N/A	Standalone tool	[168]
	Digenome-seq	Providing deep sequencing of *in vitro* Cas9-digested genomes	N/A	Standalone tool	[169]
	CRISPR-net	Quantifying CRISPR off-target activities with mismatches and indels	https://codeocean.com/capsule/9553651/tree/v1	Standalone tool	[170]
	CIRCLE-seq	Providing a sensitive and unbiased *in vitro* genome-wide off-target identification strategy optimized by using restriction enzyme for circularization of randomly sheared genome DNA	N/A	Standalone tool	[128]
Evaluation and prediction of repair outcomes	inDelphi	Predicting the mutational outcomes	https://www.crisprindelphi.design/	Web server, database	[86]
	SPROUT	Predicting the length, probability, and sequences of indels caused by CRISPR/Cas gene editing	https://zou-group.github.io/SPROUT	Web server	[113]

*Note*: DSB, double strand break; NGS, next-generation sequencing.

### Evaluation of targeted sequences by next-generation sequencing

With rapid adaptation of genome editing technology, massively parallel sequencing methods have been employed for assessing CRISPR/Cas post-experimental data. Evaluation of targeted sequences by next-generation sequencing (NGS) strategies has been developed for deeper quantification of targeted amplicon sequences. The CRISPR Genome Analyzer (CRISPR-GA) evaluates the NGS dataset and quantifies and characterizes the indels and homologous recombination events [108]. NGS also provides information regarding the selected locus, including quantification of edited-sites and other mutations detected. After scanning the reads, locating indels, and computing the allelic replacements, CRISPR-GA provides a combined report-card to the user which includes all potential information about genome editing events. Similarly, CRISPResso2 and Cas-Analyzer also provide web-accessible tools for evaluating deep sequencing outcomes of CRISPR/Cas genome editing experiments [109], [110]; CRISPResso2 also provides specific optimizations on analyzing base editing outcomes [110].

Current computation languages, such as Python and R, play a significant role in efficiency enhancement of several bioinformatic tools, which have been used to accurately detect modifications in the edited genomes by the NGS datasets. For example, “CRIS.py” is a simple and highly versatile program, which analyzes NGS data, and identifies knockout and multiple user-defined knock-in alterations from one and up to thousands of CRISPR/Cas9-edited samples [111]. CrispRVariants provides an R-based toolkit that is feasible to evaluate and visualize mutant allele types, locations, and frequency [112]. The repair outcomes of CRISPR/Cas9-generated DSBs were recently extensively studied in human primary T cells, in which Leenay and colleagues sequenced the repair outcomes at 1656 on-target genomic sites [113]; then, they used the sequencing data to develop and train a machine learning model, termed CRISPR Repair OUTcome (SPROUT). SPROUT incudes all the datasets generated from the 1656 CRISPR on-target sites and can be used to predict the length, probability, and sequences of indels generated by CRISPR/Cas9 [113]. In another study, Wang and colleagues collected 13 datasets obtained from previously reported different CRISPR/Cas genome editing experiments in six different species, including human, mouse, zebrafish, *Drosophila*, *Ciona intestinalis*, and *C. elegans*; after machine learning and featurization by eight different models, they developed an algorithm, called GNL-Scorer, for predicting CRISPR target activities [114]. GNL-Scorer, both GNL and GNL-Human, is a computational model based on the Bayesian Ridge Regression (BRR) model, which combines optimal datasets and features to address the cross-species problem. Both SPROUT and GNL-Scorer computational tools and resources will promote CRISPR sgRNA design and enhance the application of the CRISPR/Cas-based genome editing. BATCH-GE is another easy-to-use computational tool for identifying CRISPR/Cas-derived indel mutations and other precise genome editing events, including both on- and off-target impacts by analyzing huge data generated by deep sequencing technology [115], [116].

### NGS evaluation of pooled CRISPR/Cas9 libraries

Given the size and diversity of data generated by pooled CRISPR/Cas9 screens, the majority of conventional methods are not sufficient to evaluate the huge datasets generated by pooled CRISPR/Cas9 screens. To this end, several algorithms have been specifically developed for interpreting raw sequencing outputs of CRISPR/Cas9 screens, such as Model-based Analysis of Genome-wide CRISPR/Cas9 Knockout (MAGeCK) [117], caRpools [118], HiTSelect [119], Screening Bayesian Evaluation and Analysis Method (ScreenBEAM) [120], Bayesian Analysis of Gene Essentiality (BAGEL), and Permutation Based Non-Parametric Analysis of CRISPR/Cas9 screen data (PBNPA) [121], [122]. Since these analysis methods were developed for persons skilled in bioinformatics, it is difficult for many biologists or researchers with less programming background to implement them. To simplify analysis procedure, web-based interfaces have been developed to enable the users to evaluate pooled CRISPR/Cas9 screening data. CRISPRAnalyzeR is the first end-to-end analysis pipeline that integrates eight different algorithms for identification of candidate genes. In addition, CRISPRAnalyzeR is constructed in R and can be easily installed locally [123]. PinAPL-Py workflow contains various statistical models, better sequence quality checks, automated sgRNA-seq extraction, precise sequence alignment, sgRNA enrichment or depletion analysis, and gene ranking facility [124]. Its workflow can deploy a variety of well-known sgRNA libraries as well as easily upload-able custom libraries. Importantly, it can analyze the multiple CRISPR/Cas-edited experiments. PinAPL-Py ranks both sgRNAs and genes, and it provides ready-to-publish plots. However, both CRISPRAnalyzeR and PinAPL-Py have several rate-limiting steps, such as long time for raw FASTQ file transfer and complicated parameter tuning for alignment. CRISPRCloud2 employs Amazon Web Service to decrease the covert time and satisfy data-privacy requirements. Additionally, an adaptive hash-mapping algorithm was introduced into CRISPRCloud2 to increase alignment speed and accuracy [125].

### NGS evaluation of off-target effects

Off-target impact is one of the major challenges for CRISPR/Cas application in gene therapy and crop improvement as well as other areas, such as gene function studies. To reduce potential off-target impacts, many strategies have been developed, which include but are not limited to selecting high-affinity Cas enzymes, designing better sgRNAs, and using the right CRISPR/Cas reagent delivery system. However, identifying all potential off-targets is still a challenge. Identifying and quantifying unexpected genome targeting events are essential to assess the fidelity of genome editing tools as well as to guarantee the safety of gene therapeutic applications. Currently, NGS has been proved as a reliable technology to identify all potential off-target impacts as well as targeted and cleaved genome sites. However, NGS generates a vast number of reading sequences that require special computational programs to identify off-target sequences. To solve this problem, in the past several years, several research laboratories have developed computational tools that can highlight off-target activities besides the edited DNA sequences in the genome by using NGS (Table 2). Crosetto and colleagues presented a method called “direct *in situ* breaks-labeling enrichment on streptavidin and next-generation sequencing (BLESS)” that scans the DSBs at the whole-genome level by using Instant-seq software for Illumina sequencing data [126]. The efficiency of BLESS was tested in human and mouse cells by using various DSB-inducing reagents and sequencing platforms. The aforementioned method can identify telomere ends, *Sce* endonuclease-induced DSBs, and complex genome-wide DSBs. In human cells, the identified mutations (> 2000) were in the form of un-evenly distributed aphidicolin-sensitive-regions (ASRs) that was the principal proof of utilization of BLESS at the whole-genome level. Genome-wide unbiased identification of DSBs enabled by sequencing (GUIDE-seq) is an experimental approach for global detection of DNA DSBs for identifying off-target cleavage generated by Cas nucleases and potentially other nucleases, such as TALENs [69]. During identifying off-target sequences by GUIDE-seq, the authors customized a bin-consensus variant-calling algorithm based on molecular index and SAMtools; this computational program distinguishes off-target sequences from the reference sequences. This method can be used to detect off-target cleavage activities that previous computational methods or chromatin immunoprecipitation sequencing (ChIP-seq) could not detect. GUIDE-seq also detects Cas-independent genomic DSB hotspots. Giving that linear double-stranded integrase-defective lentiviral vectors (IDLVs) possesses the propensity of integrating preferentially into nuclease-induced DSBs by NHEJ repairing pathway, it has been employed to detect CRISPR/Cas-induced off-target cleavages with a very low frequency of 1% [127]. IDLVs also shows that Cas9 protein induces frequent off-target cleavages at 1-bp bulge or up to 13-bp mismatches between the sgRNA and its genomic DNA target, which may help in refining sgRNA design [127]. Circularization for *in vitro* reporting of cleavage effects by sequencing (CIRCLE-seq) identities off-targets at the genome-wide level by mapping the paired-end read sequences for searching off-target sites using *bwa mem* and *samtools mpileup.* This NGS and computational approach can be used not only for organisms with reference genome sequences but also for organisms without reference genomes [128]. However, off-target discovery methods using purified genomic DNA/specific cellular models are not capable of direct-*in*-*vivo* detection. To overcome this issue, a recently developed universally applicable approach called “discovery of *in situ* Cas off-targets and verification by sequencing (DISCOVER-Seq)” can be used to detect off-target effects *in vivo* [129]. This unbiased off-target identification approach recruits the DNA repair factors both in cells and organisms. By tracking these factors as “MRE11” [a subunit of the MRE11–RAD50–NBS1 (MRN) complex, which is tightly distributed around the Cas9 cut site], this program can detect off-target activities with single-base resolution. Moreover, DISCOVER-Seq works with several sgRNA formats and different types of Cas proteins that enable the characterization of new genome editing tools. Based on large-scale data analysis and a machine learning model, Lazzarotto and colleagues developed a “circularization for high-throughput analysis of nuclease genome-wide effects by sequencing (CHANGE-seq)” method for measuring the genome-wide activity of Cas9 *in vitro*, which includes both genetic and epigenetic impacts as well as off-target effects. Using this method, the authors identified 201,934 off-target sites from 110 sgRNA targets across 13 therapeutically relevant loci in human primary T cells [130]. From this study, they also observed that CRISPR/Cas9-induced off-target impacts were more likely to occur near active promoters, enhancers, and transcribed regions. With the rapid development of these NGS-based off-target detection approaches, more data can be produced from living therapeutic cells, which will boost the evolution of machine learning models and enhance alignment algorithms for identifying off-target impacts of CRISPR/Cas at the whole-genome level.

## Conclusion and perspectives

Given the versatility and robustness of CRISPR/Cas-based genome editing, many interdisciplinary scientists have been working to enhance this technology, including screening functionally active CRISPR/Cas nucleases, clarifying key determinants of sgRNA specificity, and reducing off-target potentials. The rapid development of computational algorithm tools accelerates greatly the quick application of CRISPR/Cas9 genome editing technology, particularly by designing optimal sgRNAs and post-genome editing data analysis. Up to now, many computational tools have been developed for designing sgRNAs and analyzing the potential on- and off-target impacts of different CRISPR/Cas genome editing systems. Certain of these programs are publicly available and have web servers for quick operation. To meet the new applications of the CRISPR/Cas systems, new computational tools for performing and analyzing CRISPR/Cas events have also been recently developed, such as scMAGeCK [131], CRISPRO [132], and ProTiler [133]. scMAGeCK links genotypes with multiple phenotypes in single-cell CRISPR screens [131]. CRISPRO maps functional scores associated with guide RNAs to genomes, transcripts, and protein coordinates and structures, which can be used to predict improved sgRNA efficacy [132]. ProTiler is used for the analysis and visualization of CRISPR screens with a tiling-sgRNA design [133]. However, there still exist several gaps in developing new sgRNA analysis tools to meet the needs of rapidly evolving CRISPR/Cas genome editing techniques.

The parameters used for building sgRNA scoring algorithms are mainly based on the data generated by CRISPR/Cas9 and CRISPR/Cas12a genome editing systems [37], [40], [89], which create targeted DNA mutagenesis via DSBs. Currently, numerous precise genome editors, such as prime editors and epigenetic editors, have been developed that are capable of rewriting genome sequences without inducing DSBs and donor DNA templates, which are especially promising tools for executing high-throughput screening and modifying base mutations [12], [134]. Given that prime editors are capable of achieving desired sequence insertions, deletions, and all 12 types of base conversions, they have been rapidly adapted in many organisms. Unlike conventional sgRNAs, the binding and sequence-specific conversion rely on an engineered multifunctional pegRNA in prime editing [61]. In addition to the common sgRNA features, pegRNAs have a programmable 3′ end, which is composed of an RT template that functions to guide DNA repair and a PBS that anneals to the nicked target DNA strand [61]. A previous study suggests that both PBS length and RT template length are important for prime editing efficiency. The suggested PBS length range is 8–15 nt, whereas RT templates are always 10–20 nt in length [12], [61]. In addition, GC content and RT template secondary structure may affect editing efficiency as well. Due to the complex combination matrix of possible PBS and RT lengths, the best method for designing pegRNAs still depends mainly on experience [12], [61]. Thus, a comprehensive study of the key determinants of the prime editing efficiency based on large-scale experimental data would be an effective approach for constructing pegRNA design tools. Additionally, as more Cas enzymes have been discovered and refined, new sgRNA design programs are also needed to work on these newly developed CRISPR/Cas systems.

Constructing sgRNA-directed mutation libraries is one of the most effective strategies to identify gene function and regulatory gene interaction networks. Current commonly used empirical algorithms are primarily derived from large-scale sgRNA analysis on human cells and the zebrafish model, but many studies demonstrate that genome editing efficiency and specificity vary widely among different organisms. Indeed, the probability of off-targets is always lower in plant species compared with animals [68], [135], [136], [137], [138], [139]. In addition to sequence features, various other factors have been identified, which affect sgRNA activity, such as chromatin accessibility, gene position, nucleosomes, and epigenomic markers [55], [140], [141], [142]. Chromatin accessibility has been demonstrated to play a dominant role in determining genome-wide binding of dCas9-sgRNA [42]. However, chromatin accessibility varies among organisms [143], [144]. Thus, comprehensive analysis of sgRNA sequence features and chromatin data across organisms might provide new insights into further optimizing scoring algorithms and computational tools.

With the quick development of CRISPR/Cas-based genome editing, it is not only limited to create targeted mutagenesis at the protein-coding region. Genome editing of upstream open reading frame (uORF) techniques provides a new viewpoint to fine-tune gene translation by means of endogenous regulatory elements. Although uORFs are found widely in eukaryotic genomes, their roles remain to be elucidated [145], [146], [147], [148]. Additionally, small RNAs are an extensive class of widespread gene regulators in eukaryotic organisms, implicated in various regulatory processes [149], [150], [151], [152], [153]. Execution of high-throughput genome-wide functional identification by genome editing of uORFs and small RNAs has a great potential to dissect the mechanisms of gene regulation. Despite the fact that a number of uORF and small RNA databases are available for a wide range of eukaryotic organisms, they are not integrated into sgRNA-designing platform. Currently there are no computational tools for designing sgRNAs for genome editing of small RNAs and uORFs. To quickly elucidate the roles of small RNAs, particularly microRNAs (miRNAs), scientists from both wet- and dry-labs should work together to develop a powerful strategy for designing sgRNAs for small RNA genome editing based on the characteristics of miRNAs, such as stem-loop structures and miRNA biogenesis [153].

The active maintenance and optimization of current computational tools is another main concern. Doench and coworkers analyzed 26,000 website-based computational tools and found that about 30% of them were inaccessible [154]. With the clarification of the mechanism underlying CRISPR/Cas binding and cleavage, the parameters on sgRNA scoring and algorithms need to be updated continuously. With the growing accumulation of experiment-based data, the existing predictive models will be further trained, which subsequently accelerates the evolution of CRISPR/Cas applications. Frequent update of currently available computational resources and tools will enhance the application of CRISPR/Cas-based genome editing.

Additionally, there are so many computational tools, including sgRNA design databases and tools for CRISPR/Cas genome editing efficiency prediction as well as on- and off-target analyses. Different tools have different advantages and disadvantages and usage for different organisms. Thus, selecting the right tool for a specific CRISPR/Cas genome editing experiment is critical. When selecting a computational tool, one first needs to know what species and even what cell types they are working on and what Cas enzymes they are using. For many cases, there are multiple computational tools that can be used; different programs may perform differently due to the fact that the different computational programs are designed based on different datasets and criteria. It is also important that further investigations uncover the causes of differences among different tools. In a recent paper, Yan and colleagues presented a way to choose a tool for designing on-target sgRNAs, and they suggest that different computational tools may be recommended in different scenarios [70]. Developing a learning-based model and also incorporating other features, such as sgRNA sequences and their structures, is the right direction for designing a good sgRNA and predicting sgRNA efficiency [70]. With the help of computational tools and resources, CRISPR/Cas-based genome editing will move forward more quickly than we thought.

## Competing interests

The authors have declared no competing interests.

## CRediT authorship contribution statement

**Chao Li:** Conceptualization, Writing – original draft, Visualization, Writing – review & editing. **Wen Chu:** Writing – original draft. **Rafaqat Ali Gill:** Writing – original draft. **Shifei Sang:** Writing – original draft. **Yuqin Shi:** Writing – review & editing. **Xuezhi Hu:** Writing – review & editing. **Yuting Yang:** Visualization. **Qamar U. Zaman:** Writing – review & editing. **Baohong Zhang:** Conceptualization, Supervision, Funding acquisition, Writing – review & editing. All authors have read and approved the final manuscript.
